# GeneDrive.jl: A decision tool to optimize biological vector control strategies under climate change

**DOI:** 10.1371/journal.pcbi.1013600

**Published:** 2025-10-21

**Authors:** Váleri N. Vásquez, Erin A. Mordecai, David Anthoff

**Affiliations:** 1 Department of Biology, Stanford University, Stanford, California, United States of America; 2 Center for International Security and Cooperation, Stanford, California, United States of America; 3 Energy and Resources Group, University of California, Berkeley, California, United States of America; University of Liverpool, UNITED KINGDOM OF GREAT BRITAIN AND NORTHERN IRELAND

## Abstract

This paper introduces GeneDrive.jl, the first software package to optimize operational planning for the biological control of mosquito disease vectors (access: https://github.com/vnvasquez/GeneDrive.jl). Mosquitoes are responsible for transmitting a significant percentage of the global infectious disease burden, a problem being exacerbated as climate change shifts the range and alters the abundance of these temperature-sensitive arthropods. The efficacy and cost of vector control varies according to species, region, and intervention type. Meanwhile, existing computational tools lack the ability to explicitly tailor interventions for local health objectives and resource limitations. GeneDrive.jl addresses this equity and efficiency gap, which is of particular concern for the tropical regions that both bear the highest mosquito-borne disease burden and are subject to disproportionate climate impacts. The software customizes vector population reduction strategies that employ genetic biocontrol, a broad suite of technologies that alter the genotype or phenotype of mosquito disease vectors, according to specific health goals and financial constraints. It can also be used to characterize risk by analyzing the temperature-responsive dynamics of wildtype vectors. GeneDrive.jl is designed to accommodate two important realities shaping the future of vector-borne disease: first, the genetic-based tools that are defining a new era in control, and second, the uncertainty that increasingly variable and extreme temperatures bring for the climate-sensitive pathogens transmitted by mosquitoes. Written in the Julia programming language, the software provides a ‘build once, solve twice’ feature wherein users may define a problem, optimize it, and subsequently subject outcomes to scenario-based testing within a single coherent platform. We demonstrate the policy relevance of this scalable open-source framework via case studies featuring the use of Release of Insects with Dominant Lethality (RIDL) to suppress *Aedes aegypti* populations in the dengue-endemic region of Nha Trang, Vietnam. This work is intended for an interdisciplinary audience and includes a Glossary to facilitate understanding (see S1 Text).

## Introduction

Tropical nations and communities bear the brunt of mosquito-borne disease, a massive global burden that saw unprecedented surges in 2023 and 2024 [1]. The annual human health impact attributed to symptomatic dengue infection is 100 million people across approximately 125 countries, while treatment worldwide is estimated to cost $8.9 billion per year [2–4]. Meanwhile, the year 2022 saw an estimated 249 million cases of malaria in 85 endemic countries; that disease costs $12 billion annually in direct costs for Sub-Saharan Africa alone [5,6]. Climate change now presents a compounding public health threat to this and other low-income regions of the world: thermal biology plays a central role in both vector population dynamics and pathogen infection and transmission from the vector [7]. A warming world is expected to increase net and new exposures to the arboviruses transmitted by the *Aedes* mosquito, while some regions will see extensions of seasonal suitability for the *Anopheles* mosquitoes responsible for spreading malaria [8–10]. By 2080, more than 60% of the global population is predicted to be at risk of dengue exposure [11]. Such projections, together with the recent severe spike in disease incidence, illustrate the importance of proactive management.

Vector population control is an important means of mitigating mosquito-borne disease; however, costs vary widely according to technology and implementation, and evolved resistance is undermining the efficacy of conventional insecticide-based methods [12]. Genetic-based innovations are recent alternatives to traditional prevention approaches; some have already been deployed in trials around the world [13,14]. Genetic biocontrol alters the genotype or phenotype of a vector species through engineering, radiation, or deliberate bacterial infection [15–19]. But the field work historically used to evaluate these methods is expensive, time consuming, and highly localized. Computational tools provide an affordable means to assess suitability for regions that may benefit from these and other technologies. Existing frameworks with varying mathematical approaches and degrees of specificity calculate the impact of environmental factors on disease vectors [20]. Others additionally permit the simulation of genetic biocontrol [21,22]. All currently available software stops short of including optimization methods to recommend mathematically feasible intervention (see Glossary, S1 Text) strategies that satisfy user requirements.

GeneDrive.jl addresses this critical gap. It takes a significant step toward enhancing efficiency and equity in vector-borne disease management as the first domain-relevant software library to include optimization capabilities, a feature that enables planners to predicate intervention decisions on regional resources and public health goals. GeneDrive.jl uses a temperature-responsive dynamic model that is comparable to previous efforts [21]. The package’s primary innovation is its decision model, a constrained nonlinear program (NLP) formulation of the temperature-responsive dynamic system (see S1 Text). Constraints include biological, climatological, geographic, and budgetary factors that restrict the feasible solution space for a potential management plan. For example, thermal fluctuations that affect vector population size may dictate the ideal timing of a given intervention, the spatial layout of a neighborhood may influence the best location to conduct that intervention, or finances may limit available intervention resources. Here, we introduce the GeneDrive.jl software design and implementation, share the results of its application to a case study based in Vietnam, and discuss its availability and future directions.

## Design and implementation

Presently, software for vector-borne disease management allows the post-hoc evaluation of intervention plans, and can be used to conduct a parameter sweep approach to intervention design [55].This method involves running multiple consecutive iterations of a simulation to evaluate a range of potential outcomes and identify the best plan possible within the scope of that sensitivity analysis. GeneDrive.jl’s decision model inverts this process to output optimal policies given defined goals and conditions as inputs (see Section 4, S1 Text).

Important earlier work has used optimal control to quantify the intervention schedules needed to achieve specified reductions in mosquito vector populations [23–25]. But these stand-alone academic analyses are not easily extended: a new problem must be formulated to accommodate each data update or alternative scenario. Obtaining such closed-form solutions also requires reducing or omitting modelled details of the ecological system [26], limiting the operational relevance of results [27]. Beyond the innovation and benefit of providing a domain-specific software framework for optimization, GeneDrive.jl’s decision model employs numerical mathematical programming (see Glossary, S1 Text) rather than analytical optimal control. This enables the inclusion of biologically detailed constraints and complex objective functions (see Glossary, S1 Text), allowing realistic and thus actionable results. It also permits scientists to rapidly, iteratively experiment with the space of possible outcomes because updates are straightforward and computationally inexpensive to re-run.

**Fig 1:**
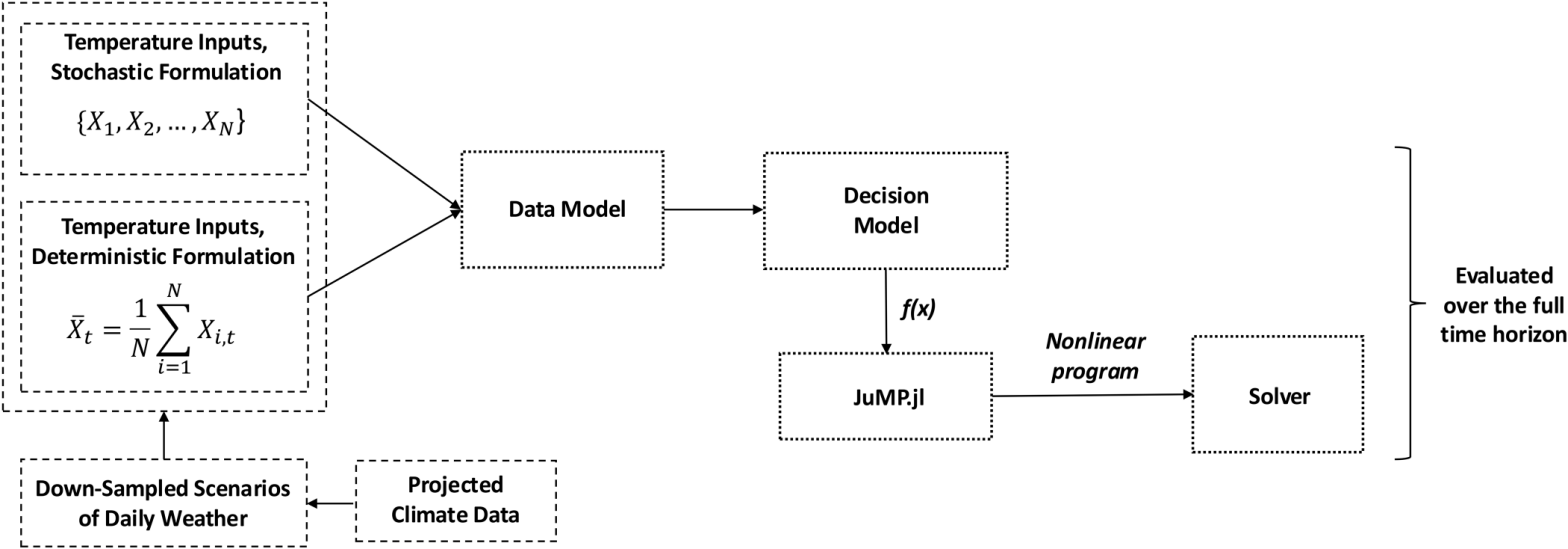
Decision Model information flow, where the set {X_1_, X_2_, …, X_N_} contains N timeseries of daily temperature and ${\overset{―}{𝐗}}_{t}$ reflects a single time series of daily temperature t, which may be an average over N time series of daily temperature X_t_. An example source of temperature information is daily weather time series down-sampled from scenarios of climate projections. Once parameterized via inputs to the Data Model, the Decision Model function f(x) is passed to JuMP.jl solvers.

A second contribution of this package is that its decision model permits stochastic optimization under environmental uncertainty (see Glossary, S1 Text). When employed in its stochastic formulation, GeneDrive.jl accounts for daily weather variability by accommodating multiple alternative temperature scenarios rather than just one average scenario. This is an important feature because averaging climatic fluctuations blunts the effect of extreme events. Such exclusion is a public health issue when climate science shows that environmental extremes can be expected to increase in frequency and intensity, and may have disproportionate impacts on vectors and transmission. By explicitly considering a range of potential weather conditions to obtain a single intervention schedule that collectively balances the potential population-level effects of various projected scenarios [28], stochastic optimization via GeneDrive.jl offers a novel and powerful tool for robust, climate-aware intervention planning. Policies produced by the stochastic decision model are more robust and adaptive to temperature-driven uncertainty, a critical concern given the thermal biology of vector dynamics and the potential for intervention efficacy to be influenced by heat in a warming world [29]. A diagram of the decision model information flow illustrates the difference between stochastic and deterministic inputs (Fig 1).

**Fig 2:**
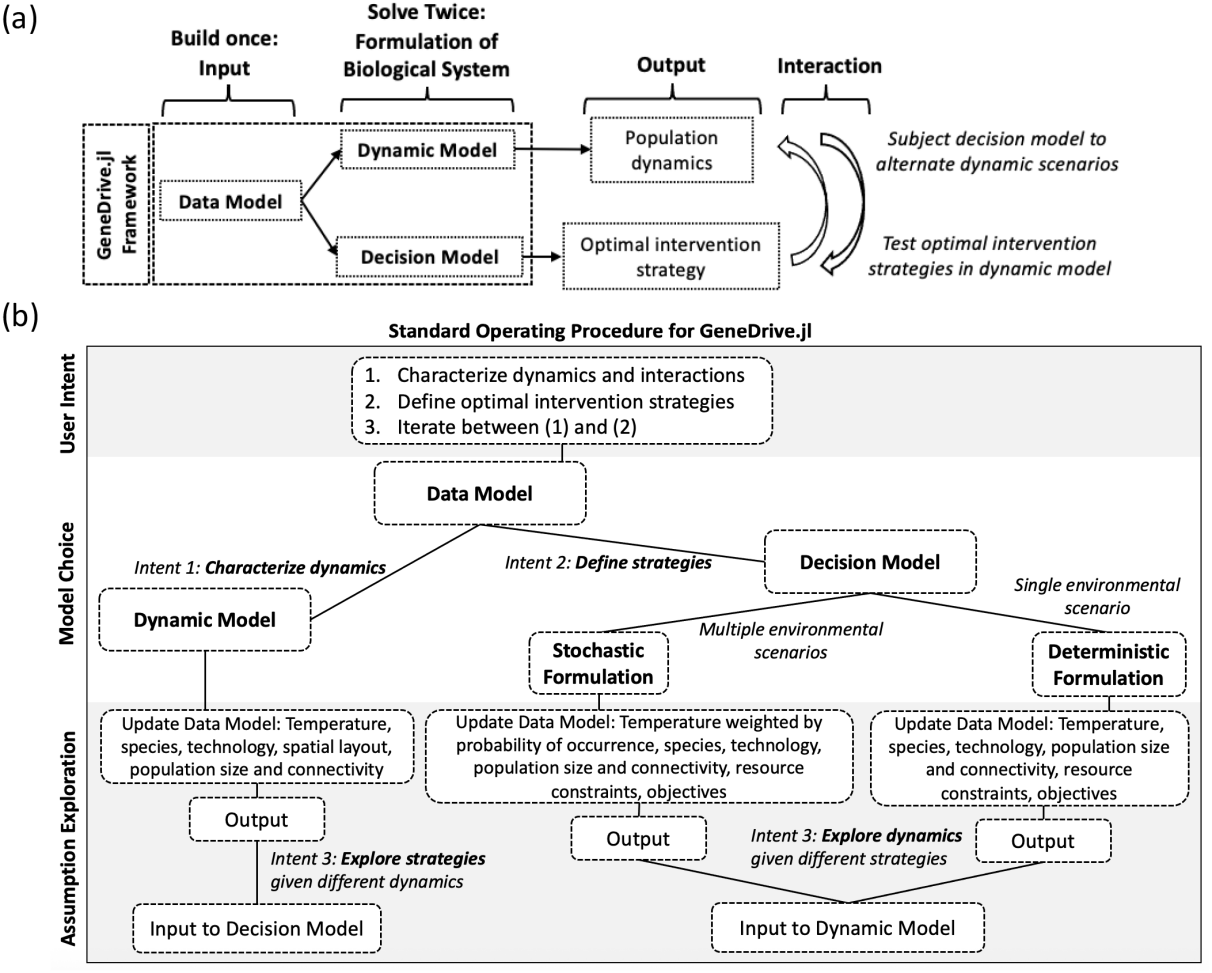
Graphical overview of and Standard Operating Procedure for the GeneDrive.jl package. Panel (a): GeneDrive.jl is a three-part framework (Data Model to standardize information inputs, Decision Model to optimize intervention strategy, and Dynamic Model to characterize ecological interactions and evaluate optimized management plans under alternative scenarios). It builds on the methodological innovations of Julia, the underlying programming language. Panel (b): Standard Operating Procedure (SOP) defined according to user intent, where the first step involves creation of the Data Model, the second step selects between the Decision and Dynamic Models, and in the third step users explore alternative parameterizations and iterate between the models as desired.

GeneDrive.jl’s dynamic model, which employs the same underlying population equations as its decision model, makes it feasible to explore prescribed intervention schedules under different scenarios (e.g., perturbed environmental regimes or alternative parameterizations of the biological system; see Fig 2 and Section 3, S1 Text) [15,29,30]. This permits users to study downstream details of the planning problem. Moving beyond intervention design, the dynamic model also allows scientists to model baseline vector dynamics absent population control, or to replicate empirical field deployments. This is useful for basic understanding of the study system. For example, it can help to characterize the behavior of interacting ecological effects. Like existing software options 55, the dynamic model may be used to conduct a parameter sweep (see Glossary, S1 Text) approach to management planning. Because the dynamic model is an ordinary differential equation (ODE) formulation of the decision model, and parameterized using the same structured data inputs (i.e., data model, see Section 2, S1 Text), it is straightforward to develop a GeneDrive.jl workflow in which optimal intervention schedules are first defined using the decision model and then evaluated under alternative assumptions using the dynamic model (e.g., under varying environmental conditions, intervention technologies, wildtype population dynamics, size, or spatial structure, etc.; see S1 Text).

Researchers as well as public health decisionmakers seeking solutions require computational tools that are easily altered and scaled. This facility – a concept sometimes called composibility (see Glossary, S1 Text) in computer science – is essential for mathematical models to evolve with new technological discoveries and changing climatic conditions. The methodological innovations offered by GeneDrive.jl build upon software design choices that prioritize composability and leverage inherent features of Julia, the software’s underlying programming language. For example, the GeneDrive.jl data model – a series of structures for standardizing information inputs – categorizes data and associates specific behavioral functions with those categories, also called Julia types [31]. One application of this approach is that the species data already included in GeneDrive.jl reflect unique thermal responses when the user declares the appropriate category, requiring no further parameterization (presently available for *Aedes aegypti* and *Anopheles gambiae*; see Section 2, S1 Text). Julia’s ecosystem of mature mathematical packages and large suite of free solvers gives GeneDrive.jl the ‘build once, solve twice’ workflow feature critical to policy design and evaluation: the dynamic model is constructed using the ODE methods in the DifferentialEquations.jl [32] package while the decision model employs the optimization methods provided by JuMP.jl [33]. An open source high-level language with C++ comparable speed, Julia also democratizes access to evidence-based planning by supplying up-to-date versions of all relevant dependencies and recording compatibility constraints via its package manager (see Section 5 and Glossary, S1 Text). This eases user experience of GeneDrive.jl by rendering a “ready-made” working environment [34]. A visual summary of the software highlights key elements of its structure and operating procedure (Fig 2).

## Results

A central result of this work is the creation of the open source GeneDrive.jl software to support the climate-aware design and deployment of biological control strategies targeting mosquito vector populations. The package offers data, dynamic, and decision models to iteratively develop management policies tailored to local contexts, accounting for unique ecological and economic realities. Table 1 summarizes the policy relevance and technical capabilities of each principal software feature.

**Table 1:** The iterative policy relevance of each software feature in GeneDrive.jl.

Model	Extensible and scalable	Simulates time- varying behavior	Optimizes for constraints and objectives	Optimizes accounting for daily weather uncertainty	Policy relevance
Data	**X**				Enhances transparency: model once, solve twice. Broadens applicability: modular updates as needed. Enables replication: stores inputs and relationships.
Dynamic	**X**	**X**			Facilitates sensitivity analyses: examine the effect of altered parameters. Allows scenario analysis: evaluate intervention impacts and tradeoffs.
Decision (Deterministic)	**X**	**X** ^*^	**X**		Permits context-relevant decisionmaking: define custom objective function as well as biological, budgetary, geographic, and climatological constraints.
Decision (Stochastic)	**X**	**X** ^*^	**X**	**X**	Accommodates uncertainty: optimize over a range of possible climates, probabilistically weighing the likelihood of each scenario.

*When no objective is defined, model dynamics are not optimized. Rather, behavior is equivalent to an ODE simulation.

*Columns highlight select package capabilities, explaining the application of each facet to policy practitioners. Rows denote whether the feature is found in the Data, Dynamic, or Decision models.*

To demonstrate GeneDrive.jl’s capabilities, we present analytical results that optimize risk reduction and cost savings goals using case studies of *Ae. aegypti*, a primary dengue vector, in the region of Nha Trang City, Vietnam [35]. Model outcomes accommodate historical observed daily temperature data for that location, as well as the ensemble median of Coupled Intercomparison Model Project 5 (CMIP5) projections of future conditions under Representative Concentration Pathway (RCP) 8.5 scenarios (the most extreme greenhouse gas concentration trajectories adopted by the Intergovernmental Panel on Climate Change (IPCC)). Each case study employs a genetic biocontrol method called Release of Insects carrying a Dominant Lethal gene (RIDL), an engineered suppression technology that eliminates competent vector populations and has been the subject of multiple field trials. We compare solutions derived using the deterministic and stochastic iterations of the decision model, and evaluate the human health and economic gains made possible by accounting for locally-specific details including daily weather variability. We also demonstrate the iterative workflow possible between the dynamic and decision models, which is conducive to adaptive policy development. Each example advances transparency by illustrating how to build a GeneDrive.jl model, input user-specific datasets, and take advantage of the sample parameters stored in pre-defined data model files.

**Fig 3:**
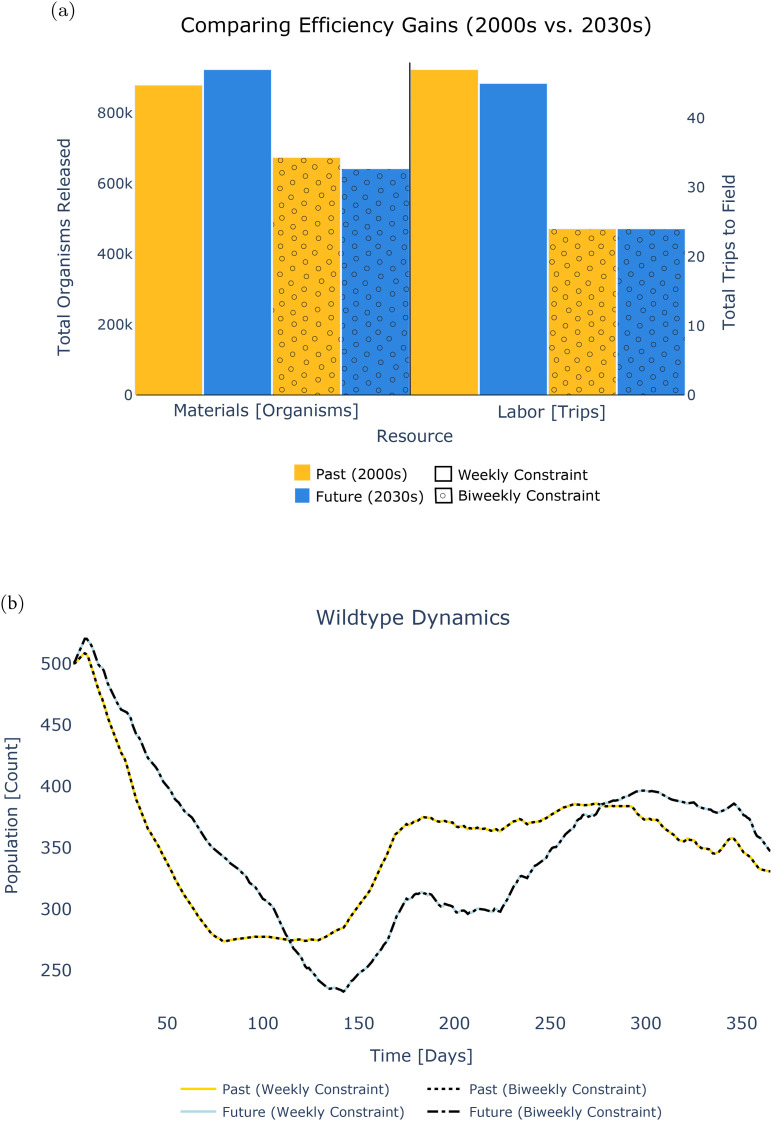
Optimized policies constrained to weekly and bi-weekly deployment under historic and future temperature regimes. (Panel (a)) shows that bi-weekly strategies allow for resource conservation in both cases while achieving the same public health goal. The resulting wildtype dynamics (Panel (b)) illustrate that optimization is useful for exploring solution space: it can accommodate necessary constraints (e.g., deployment frequency) without compromising risk reduction. Panel (a) shows that historic scenarios using bi-weekly strategies save comparably more on trips to the field, while future scenarios using bi-weekly strategies save on material costs (total transgenic organisms released). The difference in resource expenditure (materials and labor) for prescribed weekly (solid colors) and biweekly (dots) schedules under both historic (yellow) versus future (blue) regimes is shown. Panel (b) shows the resulting competent vector trajectories under each intervention and temperature scenario, with weekly (solid lines) and bi-weekly (dotted lines) releases under past (yellow) and future (blue) temperature regimes.

Optimized intervention policies shown in these results are produced by a nonlinear program that schedules the location and frequency of biocontrol deployments, along with the number of transgenic organisms that comprise each release. The deterministic program takes daily weather inputs as a single time series of temperature and the stochastic model takes a matrix of multiple time series, each of which is weighted by the probability of its occurence (Fig 3). Initially all temperature time series in the case study are assigned equal weight to illustrate the outcome of stochastic optimization. We then demonstrate how to explore specific temperature scenarios happening with higher likelihood, illustrating the notable policy benefits of this feature. Additional parameterizations input via the data model to produce the featured case studies include details of vector species, biocontrol technology, and the geographic topology across which the intervention is being optimized (see Sections 2 and 4, S1 Text). Optimization in both the deterministic and stochastic example is performed by maximizing or minimizing a customizable objective function, also called a goal (see Section 4 and Glossary, S1 Text).

All optimization results produced employ the same objective function (Equation 1). Equation 1 jointly minimizes the vector competent (wild adult female) population $F_{g}$ and the number of modifed adult male mosquitoes $c_{\hat{g}}$released to mitigate them. Variables $\hat{g}$ and g are indices of the set of genes G, respectively indicating modified and wildtype genotypes, such that the summation over g∈G in Equation 1 refers strictly to the wildtype subset of G.The minimization is sought in each daily time step t∈T, where T={1,2,…,365} and the intervention is implemented beginning at t=200. The objective is to reduce the wildtype population by a factor of 20% (ψ=0.20) with respect to its size on the first day of the simulation (t=1), from the designated date (t=200through the remainder of the problem horizon (t=365). This formulation reflects a policy-relevant suppression goal. While this work showcases a year-long period, the model may be applied to shorter or longer time frames of interest.


$$\begin{array}{c} \begin{array}{c} \underset{c_{\hat{g},t}}{\text{min}}\sum_{t\in T}\left[c_{\hat{g},t}+\sum_{g\in G}{\left(F_{g,t}-\psi F_{g,\text{1}}\right)}^{\text{2}}\right] \end{array} \end{array}$$ (1)

### Decision model: deterministic optimization

From a planning perspective, deterministic constrained optimization facilitates the design of locally tailored intervention strategies. It explores the solution space comprehensively, unlike the more widely used process of manually evaluating individually crafted policies. Optimization reduces computational effort compared to the current state of the art and assists in discovering potentially unintuitive solutions. Here, we show its application as a tool for achieving public health goals in a maximially resource-efficient manner given two sets of limitations imposed on the frequency of field deployments: weekly and biweekly. These limitations represent constraints on site accessibility, budget, or available labor. We then demonstrate the model’s utility as climate change alters the conditions under which interventions are deployed, undermining historical precedents of what works. The flexibility to quickly and cheaply optimize for shifting scenarios improves policymakers’ ability to adapt decisions even as new economic, ecological, or technological realities emerge.

A series of deterministic decision model runs show the impact that the hotter and more variable environmental conditions may have on optimal intervention schedules (Fig 3). Optimizations are conducted using a time series of average observed temperatures from the Nha Trang City region of Vietnam – chosen for its ecological and epidemiological relevance – during the 2000s, as well as an average of time series projected for the 2030s. The resulting policies produced by the model differ both in the total number of modified organisms used and the number of prescribed trips to the field. However, the vector dynamics driven by these policies are effectively identical due to the duration of the *Ae. aegypti* lifecycle, which buffers the system against differences between weekly and biweekly release schedules. We compare outputs when the solution space is constrained to weekly versus biweekly release frequencies under historical and future temperature regimes, respectively, demonstrating the relative efficiency of biweekly constraints in this instance.

Across both historic and future schedules, we observe efficiency gains when imposing biweekly operational limitations. The decrease in resource expenditure for biweekly compared to weekly deployment occurs in the historic scenario (a decadal average of annual observed temperature timeseries in the 2000s) amounts to a difference of 205,121 total organisms and 23 individual deployments. In the policies optimized for the future scenario (a decadal average of annual projected temperature timeseries in the 2030s), 281,097 fewer transgenic mosquitoes and 21 fewer release instances are employed for biweekly strategies as compared to weekly. Under weekly constraints, projected climate change will require more transgenic mosquitoes but fewer field site visits compared to historic temperatures. The difference amounts to an additional 44,171 organisms and a reduction of two discrete deployments – from 47 to 45 trips – compared to the past. Given the biweekly constraint, 31,805 fewer mosquitoes would need to be released under future projected temperatures, but the same number of trips to the field is called for in both historic and future scenarios.

### Decision model: stochastic optimization

From the perspective of resource savings for real-world practitioners, the stochastic formulation of the constrained optimization problem applied to the Vietnam example demonstrates significant advantages over the deterministic approach. We show that those material gains stand to increase with the hotter and more variable temperatures brought by climate change. The reason for this is both biological and methodological. Biologically, as environmental fluctuations grow more extreme and those extremes become more frequent there will be greater variability in the vector competent population; this demands management that is sensitive to such dynamics. Methdologically, deterministic optimization is capable of accommodating variability for one projected scenario of annual temperature at a time. However, because weather predictions carry uncertainty, it is more realistic to recognize a range of potential futures. Doing so with the deterministic model implies averaging the various potential scenarios, a characterization which would smooth the appearance of extremes. But the stochastic formulation permits consideration of multiple temperature time series in tandem, accounting for the potential outliers of each uncertain projection and returning an optimized policy that balances the possibility of their occurance.

**Fig 4:**
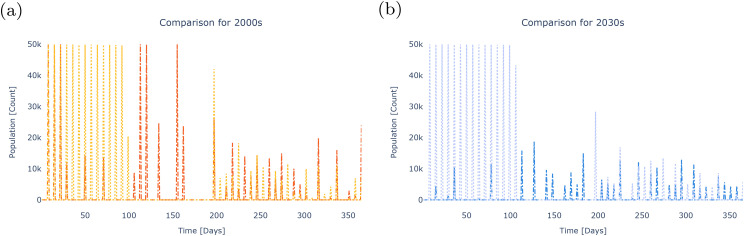
Comparing deterministic and stochastic optimized policies under historic (Panel (a), 2000s) and future (Panel (b), 2030s) temperature regimes shows that cost savings are significantly larger when applying the stochastic decision model to define future strategies. These gains are demonstrated both in amount of material used and total trips to the field. Panel (a) shows deterministic (yellow) and stochastic (orange) schedules given historic temperatures. Panel (b) shows deterministic (light blue) and stochastic (dark blue) schedules given future temperatures.

As shown here, the stochastic decision model yields biocontrol strategies that employ hundreds of thousands fewer modified organisms across both historic and future scenarios (Fig 4). Policies produced by the stochastic program reduce trips to the field over the course of both historic and future modelled years by approximately 30% compared to deterministic optimization. The value of accounting for the daily weather uncertainty of six observed and projected time series of annual temperature in the Nha Trang City region is illustrated by comparing stochastic decision model outputs (darker colors) to deterministic decision model results (lighter colors) for each regime (Fig 4). Schedules are optimized for the historic (2000s) and future (2030s) period (Fig 4a and 4b, respectively). When assessing the prescribed change in total numbers of released organisms, savings enabled by the stochastic compared to the deterministic model are 284% in the future case study, compared to 45% in the historical example. Thirty-two deployments are prescribed by the stochastic model under both historic and projected temperatures, compared to the deterministic model policy of forty-seven (historic) and forty-five (future).

### Dynamic model: characterizing outcomes

After defining optimal intervention strategies using the decision model, we use the dynamic model of GeneDrive.jl to simulate them and examine their effect on mosquito populations over time under different climate scenarios. This illustrates the tradeoffs of alternative policy options relevant to wild *Ae. aegypti* populations whose dynamics are responsive to four different temperature regimes: two with high daily thermal variability and two with median daily thermal variability (see S1 Text). Results highlight that as temperatures become hotter and more variable, it will be increasingly important to analyze potential compromises between the economic cost and feasible health outcomes of vector-borne disease interventions, because these tradeoffs may become more stark.

**Fig 5:**
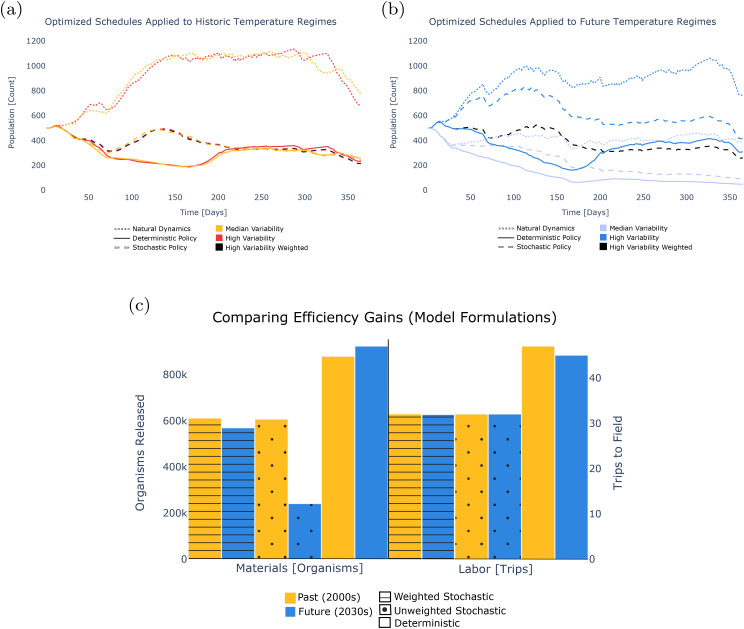
Population dynamics subjected to alternative intervention policies demonstrate that deterministic strategies perform well under historic temperature scenarios by achieving comparable health outcomes with fewer resources, while stochastic strategies provide superior public health protection under uncertain or highly variable future climates. Panel (a) shows the suppression in natural vector population dynamics (dotted lines) resulting from policies determined by stochastic (dashed lines) and deterministic (solid lines) optimization, where yellow indicates median temperature variability, orange indicates high temperature variability, and black highlights the outcome of the weighted stochastic policy under the high variability scenario. Panel (b) again shows the suppression in natural vector population dynamics (dotted lines) resulting from stochastic (dashed lines) and deterministic (solid lines) policies, where light blue indicates median temperature variability, dark blue indicates high temperature variability, and black highlights the outcome of the weighted stochastic policy under the high variability scenario. Panel (c) shows deterministic (solid colors), probabilistically weighted stochastic (lines), and unweighted stochastic (dots) resource expenditure given past (yellow) and future (blue) temperature scenarios.

Under historical temperature regimes, optimized deployment schedules – whether produced via stochastic or deterministic optimization – reduce the *Ae. aegypti* population to comparable levels (Fig 5a). High versus median temperature variability scenarios do not produce significantly different results in this case study. When employing the stochastically-generated intervention policy, high variability scenarios briefly lead to elevated risk levels (a larger vector competent population). More heavily weighting the probability of the temperature scenario featuring the highest daily variability (see S1 Text) does not generate dynamics that are distinct from when all scenarios are weighted equally. From a health policy perspective, these results suggest that the deterministic strategies may be a pragmatic choice, assuming historical temperature scenarios. Stated resource constraints (material quantity and frequency of trips to the field) are met while achieving greater immediate health outcomes in the short term and approximately equivalent outcomes in the longer term compared to stochastic strategies.

Under future temperature projections with median daily variability, deterministically-defined interventions are 46.9% more effective than unweighted stochastic strategies in suppressing the vector population by the end of the observed period, but at a much higher resource cost (Fig 5b). These results exemplify how the dynamic model furnishes policymakers with additional decisionmaking context: there is a bigger price tag incurred by the more resource-demanding deterministic release schedule; however, as evidenced by the dynamic model results, this strategy reduces the potential human health and consequent downstream economic costs compared to the unweighted stochastic release schedule, in which a larger disease-competent mosquito population remains. When the future scenario with the highest variability is probabilistically weighted (black dashed line in Fig 5b) to reflect the assumption – supported by climate science – that higher variation in daily temperatures will become more likely in the future (see Section 1, S1 Text), the competent vector population at the end of the simulated year is 18.9% lower than under the deterministic model strategy (dark blue solid line in Fig 5b). Decisionmakers must consider such gains while taking into account the resource expenditures prescribed by different model formulations (Fig 5c).

## Availability and future directions

We demonstrate the value of constrained optimization for biocontrol purposes and characterize the relative benefits of deterministic and stochastic decision models for policy applications, highlighting how each integrates with dynamic simulations of temperature-sensitive vector ecology. This work is directly responsive to the expert-expressed need for software decision tools that facilitate operational planning for climate-sensitive diseases [20]. We show that a deterministic nonlinear program produces health-effective and cost-efficient risk reduction assuming historical scenarios of daily temperature in the region of Nha Trang City, Vietnam, and that under the same historical climatic conditions a stochastic nonlinear program achieves nearly equivalent results while expending fewer resources in both labor and raw materials. The results of our stochastic decision model applied to future temperature projections show that, when high daily variability is more likely to occur, assigning greater likelihood to those scenarios yields intervention policies that reduce public health risks more effectively than those that treat all projected scenarios equally. Box 1 provides additional examples of key questions to which GeneDrive.jl might be applied.

**Box 1: Key questions to which GeneDrive.jl might be applied.**

Select examples of research or practice-oriented questions that could be addressed using the current iteration of the software include:

- Dynamic Model: Characterize the seasonal population fluctuations of mosquito disease vectors (e.g., *Aedes aegypti, Anopheles gambiae*) absent intervention, given alternative thermal scenarios.
  - For example, how might specific species dynamics change under future temperature projections for Peru? For different regions of Brazil?
- Decision Model: Design locally targeted vector population management plans to address ongoing outbreaks or minimize future disease risk while remaining within a defined budget (e.g., materials, labor, schedule).
  - For example, what control strategy is optimal for the city of Singapore, given its unique goals and resources? How does this strategy compare to that recommended for the city of Dhaka, with its distinct geographic features, goals, and resource availability?
- Iterating between the Dynamic and Decision models: Compare the efficacy of technological interventions (e.g., suppression approaches using Release of Insects carrying a Dominant Lethal gene (RIDL) or *Wolbachia* infection, replacement approaches using Homing Gene Drive (HGD) or *Wolbachia* infection) under different temperatures, spatial topographies, and vector population size.
  - For example, does network connectivity inform whether RIDL or Wolbachia succeeds sooner in reducing vector populations? Is effectiveness impacted by a tropical versus a temperate climate?

The case studies presented here incorporate a critical exogenous stochasticity (i.e., temperature variability). However, it is also important to consider environmental uncertainties additional to temperature, such as precipitation [36]. Future work will expand the GeneDrive.jl model to accommodate more extensive uncertainty quantification, including with respect to the mechanistic modeling of vector species and genetic-based intervention technologies.

While some of this work is underway, advanced users and those who seek to contribute to the development of GeneDrive.jl may leverage its modular design to extend or add to existing capabilities (see S1 Text). For example, the current framework operates as an open-loop, but incorporating closed-loop methods could enable re-optimization based on updated forecasts or entomological surveillance data, enhancing responsiveness to real-time environmental variability. Methodological limitations inherited from the underlying GeneDrive.jl vector dynamics model, such as the omission of potential temperature-driven behavioral adaptations (e.g., mosquito heat avoidance), the assumption of spatial homogeneity within geographic nodes, and structural choices in the parameterization of temperature-sensitive life history traits within the ODE framework, present further opportunities for refinement. For further detail, see Vásquez et al. (2023).

The optimization case studies presented are also subject to simplifying assumptions which future work might usefully address. For instance, economic and logistical costs are modeled in generalized terms; incorporating empirical data on region-specific cost structures could improve practical relevance. In addition, budgetary uncertainty is not yet incorporated into the stochastic formulation. Resolving such limitations could help bridge the gap between computational tools and operational decision-making in resource-constrained settings. Box 2 provides a discussion of GeneDrive.jl in policy context.

The GeneDrive.jl software is located on GitHub at https://github.com/vnvasquez/GeneDrive.jl. All code and data inputs required to reproduce the work in this paper is included in the https://github.com/vnvasquez/paper-genedrivesoftware repository and additionally available on Zenodo (https://doi.org/10.5281/zenodo.17353504). Model dynamics are validated against empirical study results in Vásquez et. al. (2023). This work contributes the first open-source software for optimizing genetic biocontrol strategies under climate uncertainty, helping to bridge a critical gap between ecological modeling, operational planning, and public health decision-making.

**Box 2: GeneDrive.jl in policy context.**

Uncertainty in daily weather already presents a challenge for the design and implementation of biocontrol for public health. As temperatures warm and become more variable, the complexities inherent to mitigating mosquito-borne diseases – including the dengue virus explored in our case study – will continue to grow. For example, the field trials upon which current intervention planning is largely based will provide progressively less reliable guidance for tuning future deployment schedules. Vector-borne disease management thus stands to gain from computational methods, including the simulation approaches already in use, which incorporate mechanistic knowledge based on empirical experiments. GeneDrive.jl additionally illustrates the importance of constrained optimization for vector control purposes. The hyper-local nature of mosquito-borne disease is driven by socioeconomic, environmental, and biological factors: to develop public health interventions that are regionally appropriate, these constraints must be taken into account explicitly. As demonstrated by multiple industries, including within the broader health domain [36–39], optimization via mathematical programming is a means of doing this while enabling the straightforward, iterative examination of alternative policy goals.

The intensity and frequency of heatwaves have been found to negatively impact the performance of at least one genetic-based intervention method [29], further highlighting the need for planning models that account for temperature-dependent dimensions of uncertainty. GeneDrive.jl showcases how daily temperature, including characteristics such as median versus high thermal variability, affect vector population dynamics and control. This exemplifies the utility, unique to this software library, of including a dynamic and decision model in a single coherent package: together, they facilitate a more comprehensive understanding of the conditions that contribute to differences in prescribed interventions as well as the potential range of outcomes from those policies. This can inform a richer evidence base for the discussion of optimized biocontrol. However, bridging the divides that presently exist between model outcomes and practitioner discussion requires developing more robust pipelines for collaboration [40] . The roadmap that enables simulation results to guide policy implementation may differ regionally, and while recent efforts have begun establishing communities of practice to address this challenge [20]. much work remains.

One step toward enhancing the policy relevance of software such as GeneDrive.jl is using it to design and evaluate a future field trial, such that results can be assessed using contemporaneous empirical data to evaluate the potential effect of optimized schedules informing adaptive deployments. Another avenue for increasing the accessibility of software tools and allowing form to suit necessary function is to develop useability studies that include bench and field scientists as well as policymakers. This would challenge the current paradigm of models being largely academic exercises. The need for improved participatory approaches is not, however, limited to collaborations between modelers, scientists, and decisionmakers: community consultation is also a key component of ensuring that software design answers non-expert concerns, examples of which might include producing estimates of intervention technology risk or options to simulate and optimize remediation (see Glossary, S1 Text) of genetic biocontrol.

## Supporting information

S1 Text Supplementary information. (DOCX)

S1 Fig Abstract tree hierarchy illustrating the structure of data model components. Arrows with dotted lines indicate optional selections, and those with solid lines indicate components that are required to fully specify and store or run a GeneDrive.jl problem.
(TIFF)

S2 Fig Dynamic model information flow. (TIFF)

S3 Fig Decision model information flow. (TIFF)
