# Supplementary material for: GeneDrive.jl: A decision tool to optimize biological vector control strategies under climate change
Source: PLoS Comput Biol. 2025 Oct 21;21(10):e1013600. doi: 10.1371/journal.pcbi.1013600 (PMC12551955; doi:10.1371/journal.pcbi.1013600)
Supplement: S1 Text — (DOCX) [file pcbi.1013600.s001.docx]

*Section 1: Climate data*

Historical daily temperature records for the region of Nha Trang City (Nha Trang), Vietnam (latitude 12.2534, longitude 109.1871) are from the Global Historical Climatology Network (GHCN) database maintained by the National Centers for Environmental Information of the United States National Oceanic and Atmospheric Administration (NCEI-NOAA). Two villages in the Nha Trang region, Vinh Luong Ward (Vinh Luong) and Tri Nguyen Village (Tri Nguyen), are the sites of recent genetic-based interventions for *Ae. aegypti*. Lacking weather data from the villages themselves, subsequent analysis of deployments used daily records from Nha Trang.^1^ Future heatwave data under Representative Concentration Pathway (RCP) scenario 8.5 was provided by Dong et. al. (2021).^2^ These data were used to develop projected heatwave scenarios for Vásquez et. al. (2023) using historical temperature data for the baseline period (1990-2005) as well as Coupled Model Intercomparison Project Phase 5 (CMIP5) projections of 2030 and 2050 temperature deltas for Vietnam from the World Bank Climate Change Knowledge Portal, which provides downscaled ensemble means derived from up to 30 CMIP5 models.^3^ See Vásquez et. al. (2023) for a thorough description of the anomaly method used to create the heatwave scenarios.

*Section 2: Data model*

The data model is designed to compose each GeneDrive.jl problem using broad thematic components (e.g., temperature data, organism data). It stores the details of interest, enforcing consistency in the specification of data across computational experiments to facilitate both reproducibility and data sharing. ^4,5^ Each thematic component includes details that are themselves encapsulated in subcomponents (S1 Fig). This modularity promotes code re-use as different research questions or focal areas are studied; the functional form or parameterization of each component may be independently altered per problem specification without changing the larger code base.^6^ These structures grouping related variables, which can include different data types such as integers, floats, and strings, are also called “structs.”

Experimentation with different environmental assumptions is conducted by parameterizing one of three `Temperature` structures. These include `ConstantTemperature` to specify a static thermal environment, `SinusoidalTemperature` for an idealized, seasonally fluctuating regime, and `TimeSeriesTemperature` in which vectors of daily values can be stored. Each of these descriptions of thermal trend can be accentuated with heatwaves and cold snaps using the `TemperatureShockData` structure to impose time-bound increases or decreases in temperature.

The `Organism` type in GeneDrive.jl includes a `LifeStages` data container for details of species-specific development time and mortality rate for juvenile and adult mosquito life stages. For select species (presently the primary vectors of dengue and malaria, respectively: *Ae. aegypti* and *An. gambiae*), empirically derived functions are included to characterize vital rate responses to environmental perturbations such as temperature. Static parameter values may also be used. The `Genetics` data type within the `Organism` component defines the likelihood with which offspring will be produced, as well as fertility rates, sex ratios, and varied fitness costs (e.g., the degree to which fecundity for modified organisms is biased with respect to their wild counterparts). It denotes the properties of the biocontrol technology being deployed (presently, options include Release of Insects carrying a Dominant Lethal (RIDL), *Wolbachia*, and Homing Gene Drive (HGD)).

For geographic information, the GeneDrive.jl data model provides a `Node` and a `Network` structure. The `Node` datatype includes fields to store the `Organism` and `Temperature` components as well as geographic coordinates and location name. A `Network` structure is comprised of a collection of `Node`s. Each species, life stage, genotype in a `Node` is assigned a rate of dispersal using a transition matrix. Nonzero rate values instantiate bidirectional `Node` connections. This feature enables users to account for a diversity of demographic and genetic migration tendencies as well as exogenous factors that may transport organisms from location to location.^7–9^

Anthropogenic actions such as biological control may be studied in the GeneDrive.jl dynamic model using the `Release` object. This data type defines the time at which a certain number of organisms are added to or removed from a population during dynamic simulation. When specifying an intervention schedule, fixed or variably sized releases can occur at flexible intervals. Alternatively, interventions may be conducted in an adaptive manner that directly accounts for the size of the standing wildtype population using `ProportionalRelease`, wherein the release size is defined according to its relative magnitude with respect to the population of interest at a given timestep (e.g., 15% of wild females present on day 100 of the simulation).

For computational experiments that optimize anthropogenic interventions, the `ReleaseStrategy` structure stores the information about operational requirements and limitations that is used in the decision model. The structure name is a nod to the biocontrol intervention strategies commonly used in the public health, agriculture, and invasive species arenas wherein organisms modified for, for example, sterility are released into the environment to reduce the standing wildtype population of a given species.^10^ The values of this structure are assigned on a per-node basis in the case of a network implementation, enabling spatially explicit constraints and the exploration of myriad policies. Default values are supplied where no information is specified; this information is accessible by viewing the fields of the data model.

The case study presented in this work illustrates construction of the GeneDrive.jl data model (Code Block 1). It defines the vital rates, thermal biology, genetic characteristics, and habitat of an *Ae. aegypti* mosquito. The data model in this example is largely built by drawing from pre-constructed information stored in the GeneDrive.jl library. This highlights the code brevity and simplicity made possible by the software’s design, particularly once problem information has been assembled and saved. All data used in this case study are available in the package for replication and extension.


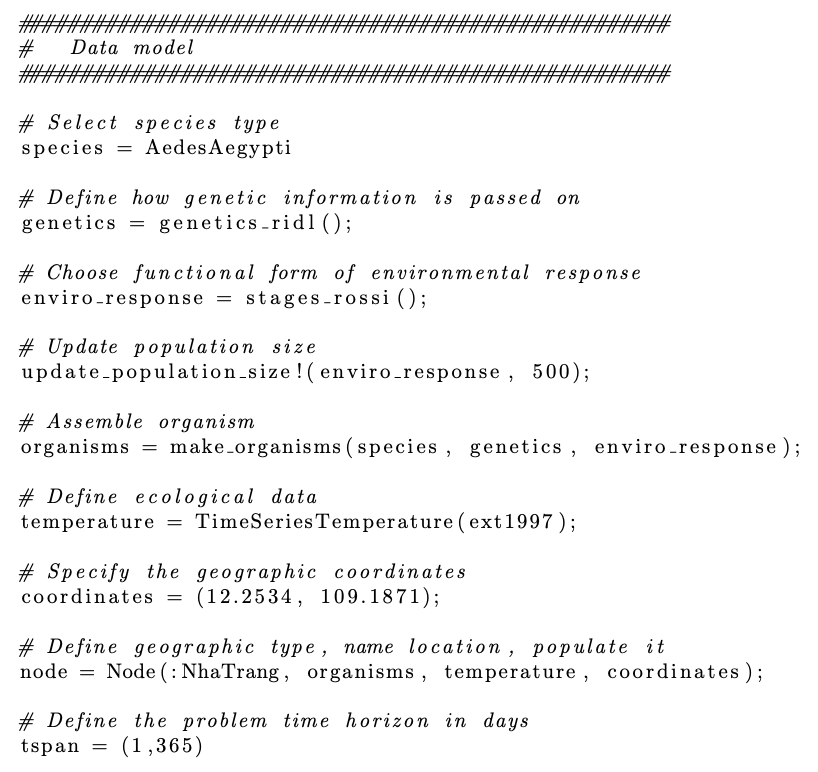


***Code Block 1:*** *Creating the data model. Find an executable version of these code examples in the replication code at https://github.com/vnvasquez/paper-genedrivesoftware, file `codeblocks.jl`. Find temperature data in the replication code file `data.jl`.*

*Section 3: Dynamic model*

The dynamic model in the GeneDrive.jl framework is a system of ordinary differential equations (ODEs) that simulates mosquito population dynamics (Supporting Information Equation 1).


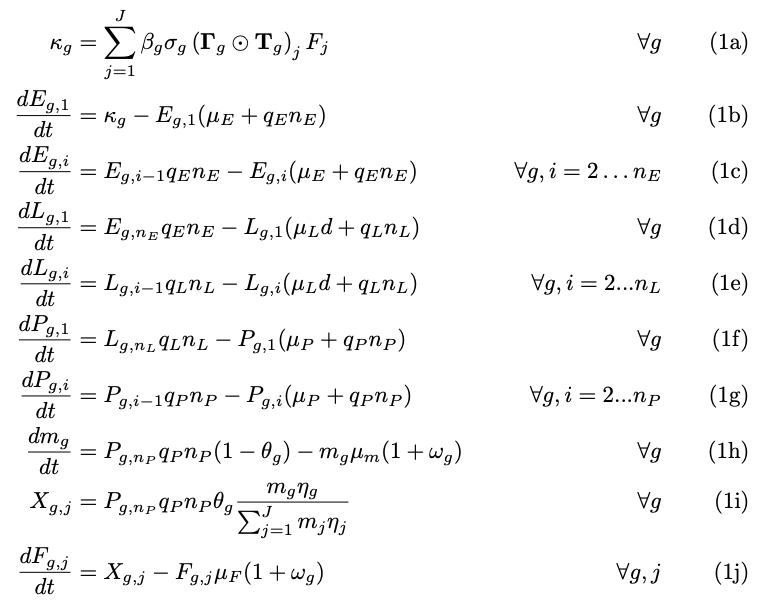


Each geographic node contains equations of motion (SI Eq.1a-1j) calculating oviposition ($\kappa$), the presence of juvenile life stages egg (*E*), larva (*L*), and pupa (*P*), and adult life stages male (*m*) and female (*F*). Adults are assumed to mate a single time; the variable *X* represents the inflow of newly mated females into the stock variable *F*. The organisms within a shared life stage and node are parameterized by the same death ($\mu$) and maturation (*q*) rates.^11^  Larval stage mortality is additionally regulated by logistic density dependence ($d=1+ \sum(L)/k$, where $k$ represents carrying capacity). Following multiple examples in ecology and public health applications, we use Erlang distributions (*n*)^12–14^ to incorporate flexible dwell times helpful for modeling the environmentally-influenced life stage duration of juvenile mosquitoes.

Juvenile life stage variables are indexed by genotype (*g*) to reflect a particular pattern of inheritance and by Erlang substage (*i*) to model dwell time during development. Adult female life stages are indexed by their own genotype (*g*) and that of the adult male (*j*) with whom they have mated. All variables are implicitly indexed by time; for brevity of presentation we omit these in Eq. 1. In the case study examples presented by this work, *g* differentiates between RIDL-modified and wildtype individuals, while mortality rates *μ* and development rates *q* are dynamically calculated for each life stage using the *Ae. aegypti*-specific temperature-sensitive functional forms developed by Rossi et. al. (2014).^15^ Fitness cost of the RIDL technology is parameterized with mid-range estimates from field trial data,^16^ with transgenic males penalized by a 3.1% decrease in mating fitness (${}_{g}$) and an 18% additional increase ($\omega_{g}$) in mortality. The values used for all parameters in the case studies presented by this work are stored in GeneDrive.jl data models that ship with the library. Genetic and life stage values, respectively, can be accessed directly by running genetics_ridl() and stages_rossi().

Parameter *β_g_* represents female fecundity, while *σ_g_* represents male fecundity. *Γ_g_* and *Τ_g_* are genotype-specific inheritance and survival probability, respectively; the Hadamard (element-wise) product of these matrices in SI Eq.(1a) gives the joint probability of inheritance and survival for genotype *g*. Parameter $\theta_{g}$ dictates the male to female offspring emergence ratio. Values for *Γ_g_* used in the worked examples are from Sánchez et. al. (2020)^17^; the formulation of SI Eq.(1a) and (1i) follow Sánchez et. al. (2020).^18^ The ODE system is initialized at equilibrium by setting the left hand side to zero and solving (see Supporting Information Section 5: The Julia advantage).

While the data model establishes an experimental record of the parameters used to initialize a dynamic simulation in GeneDrive.jl, the solver permits updates to these values over the course of a dynamic model run (see Supporting Information Section 5: The Julia advantage). This feature facilitates investigating the effect of system perturbations. It is enabled by the information flow that characterizes the dynamic model (S2 Fig). In the examples shown, a use case is the introduction of RIDL-modified organisms into the modelled population at the various timesteps dictated by different optimized policies (see Results). Other work has applied this capability to alter the environmental conditions to which simulated organisms are responding.^19^

The information flow key to such computational experiments is enabled by modular design (see Supporting Information Section 5: The Julia advantage). Modularity also enforces the separation of simulation and solving algorithms, curtailing the need for extensive code rewrites when models are updated and run. For example, to create the dynamic model once the data model is built, all that is required is solver selection followed by simulation using the `solve_dynamic_model` API (Code Block 2). Outputs may then be pre-processed for analysis using `format_dynamic_model_results.` Modular design likewise permits solution methods to be easily exchanged, allowing the exploration of alternative backend solvers to improve performance. The dynamic model examples shown as case studies use Tsit5, an explicit Runge-Kutta method recommended for most non-stiff systems.^20^ However, there are a robust suite of options compatible with or included in the DifferentialEquations.jl platform upon which the GeneDrive.jl dynamic model is built (see Supporting Information Section 5: The Julia advantage).


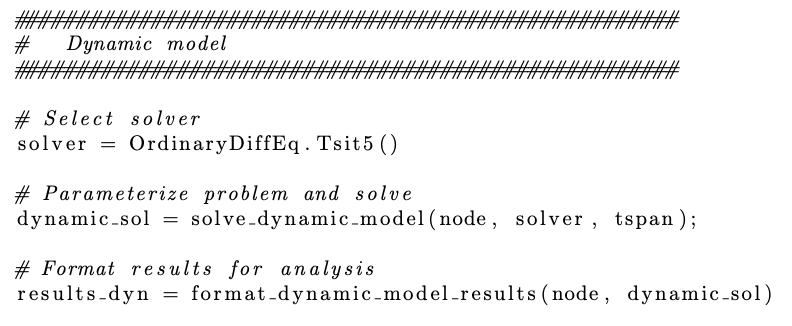


***Code Block 2:*** *Creating and solving the dynamic model, then formatting outputs for further analysis, is straightforward in GeneDrive.jl. Find an executable version of these code examples in the replication code at https://github.com/vnvasquez/paper-genedrivesoftware, file `codeblocks.jl`.*

*Section 4: Decision model*

The GeneDrive.jl decision model is a nonlinear program (NLP) formulated by discretizing the dynamic model’s system of ODEs using Euler approximations with a daily timestep. It determines the optimal set of control (also called decision) variable values in pursuit of a given objective (goal). The objective is a function that maximizes or minimizes, and can be a measure of performance, cost, or any other metric of interest. The “best” set of decision variables to satisfy an objective function are often referred to as the optimal schedule or optimal policy and are subject to constraints. Constraints, or restrictions on the feasible solution space, are defined using mathematical equalities or inequalities.

The equality constraints of the GeneDrive.jl optimization problem are the discretized system of ODEs representing the vector population. Thus, they inform the feasibility of a given simulation with biological details. Constraints representing non-biological or operational limitations, such as resource availability, enter the decision model as inequality constraints. These are assigned on a per-node basis using the data model, enabling the exploration of spatially differentiated policy combinations. Like the dynamic model, decision model parameters are species-specific and populated by data model values. Unlike the dynamic model, solution methods evaluate problem information over the full time horizon rather than at each timestep (S3 Fig).

The default nonlinear solver in GeneDrive.jl is the Interior Point OPTimizer (Ipopt),^21^ a free software library for largescale optimization. This choice may be updated by users interested in exploring different solvers or augmenting the Ipopt optimizer with additional algorithms to accelerate solutions or improve accuracy. Ipopt internally utilizes a linear solver. Various free and commercial linear solvers can be chosen from among a list in the Ipopt online documentation^21^ that are compatible with the JuMP.jl platform used to create the GeneDrive.jl decision model (see Supporting Information Section 5: The Julia advantage). While sample objective functions are supplied in the GeneDrive.jl package, optimization goals are unique to package users and thus also customizable.

Again, modularity simplifies both problem specification and simulation, maximizing code re-use. After populating the `ReleaseStrategy` structure with context-specific operational constraints and assigning those values to the `Node` structure of interest in the data model, a solving algorithm is selected. The deterministic optimization problem is then parameterized using `create_decision_model`, solved using solve_decision_model_scenarios, and results formatted for analysis with `format_dynamic_model_results` (Code Block 3, left panel). The stochastic optimization model is differentiated via the form of temperature structure inputs to the updated data model: it takes a matrix of time series together with associated probabilities, unlike the single time series used as the input to the deterministic optimization (Code Block 3, right panel). Operational constraints specific to the experiment of interest in this case study permit only male organisms modified using the RIDL technology to be released, with the timing of those deployments allowed weekly in batches numbering 50,000 organisms or less at each release.


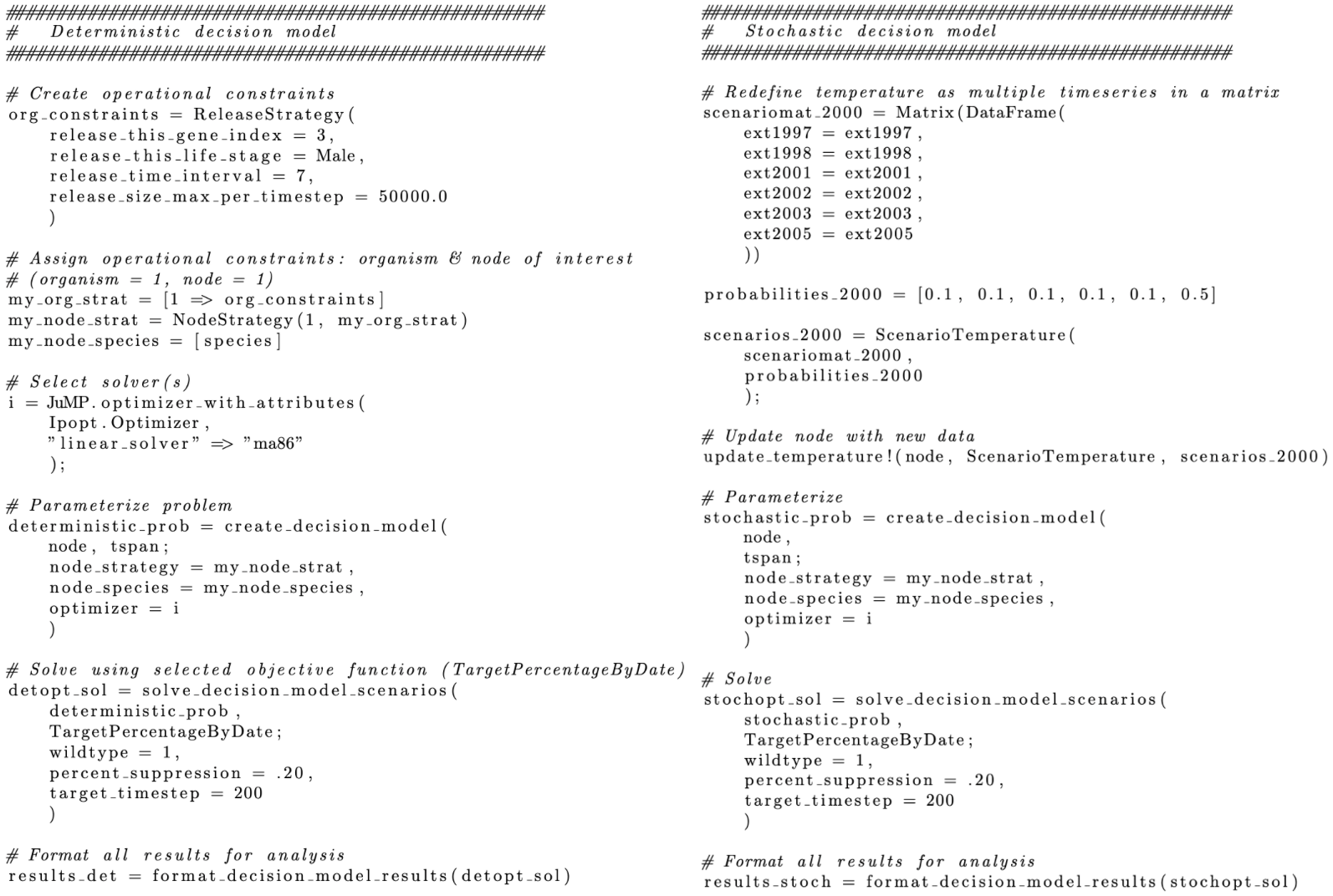


***Code Block 3:*** *Creating the deterministic and stochastic optimization models. Find an executable version of these code examples in the replication code at https://github.com/vnvasquez/paper-genedrivesoftware, file `codeblocks.jl`. The custom function `TargetPercentageByDate` is defined in the file `data.jl`, which also includes the temperature data referenced in this code block.*

*Section 5: The Julia advantage*

The entire GeneDrive.jl stack is free and open source, lowering barriers to usage and enhancing the transparency of its methods and outputs. This is enabled by the Julia scientific programming language, as well as the packages within that ecosystem used to develop this work. The equilibrium of the dynamic and decision model systems is found using NLSolve.jl,^22^ a library for solving nonlinear equations. The dynamic model itself is built upon DifferentialEquations.jl,^20^ a suite for numerically solving differential equations that also furnishes efficient differential equation solution methods. It is DifferentialEquations.jl that allows the use of callback functions within GeneDrive.jl. This means that custom changes can be made to data values or functions at specific points during the execution of a simulation, permitting users to model exogenous inputs to the system including the addition of new species and the manifestation of heatwaves. The decision model is developed using JuMP.jl,^23,24^ a domain specific modelling language (DSL) and collection of supporting packages for mathematical optimization that is embedded in Julia. Finally, Julia supports a growing community of developers interested in biological questions and expressly focused on cutting edge advances in scientific programming.^25,26^

**Glossary**

| **Term** | **Definition** |
| --- | --- |
| Composability | A property of software components or modules that promotes their capacity to be independently developed, combined, and reused within larger systems. |
| Compatibility constraint | Common standards or protocols that ensure different software can work together smoothly. |
| Constraint | A condition or limitation that defines the allowable range or relationships among variables, restricting the possible solutions in a problem. |
| Data model | A conceptual representation of how data is structured, organized, and accessed within a system. It defines the relationships between different types of data and the rules for storing, retrieving, and manipulating it. |
| Decision model | A mathematical representation of a problem that identifies the best course of action among various alternatives, given objectives (goals), constraints (limitations), and decision variables (choices or actions). |
| Dependency | A relationship between software components, modules, or entities, where one relies on the other for some functionality, data, or resources. |
| Deterministic optimization | A mathematical method for finding the “best” (“optimal”) solution to a problem according to a set of rules or algorithms, without considering randomness or uncertainty. |
| Density dependence | The variation in the growth or survival of a population as dictated by the size of that population relative to environmental carrying capacity. |
| Dynamic model | The mathematical representation of how a system changes over time, using equations (in this work, Ordinary Differential Equations) to capture the relationships between variables and their rates of change. |
| Genetic biocontrol | As used in this work, a broad suite of technologies developed via engineering, radiation, or deliberate bacterial infection that alter the genotype or phenotype of mosquito disease vectors by suppressing or wholly replacing vector populations. |
| Intervention | As used in this work, any action or measure aimed at improving health outcomes or preventing disease. Here referring specifically to genetic biocontrol methods for the mitigation of mosquito-borne illness (i.e., release of modified or transgenic vectors). |
| Mathematical programming | A method often used in operations research and engineering that employs discrete decision variables to optimize mathematical models subject to constraints; it involves numerical techniques. |
| Nonlinear program | A mathematical programming problem where the objective function or constraints are nonlinear. |
| Objective function | The goal that an mathematical optimization algorithm seeks to maximize or minimize. Also called a “cost function” in economics. |
| Open-loop | An optimization approach where decisions are made for the full time horizon in advance, without updating based on new data during implementation. This is in contrast to closed loop optimization, an adaptive approach where decisions are updated during implementation based on new data or feedback, enabling real-time adjustment to changing conditions. |
| Optimization | The process of finding the best solution from a set of feasible options (solution space), seeking to maximize or minimize an objective function (goal) while satisfying stated constraints (limitations). |
| Optimal control | An optimization method method that employs continuous decision variables and focuses on finding the best control policies for dynamic systems over time; it involves analytic techniques. |
| Oviposition | The process by which a female organism (here, mosquito) deposits or lays eggs. |
| Parameter sweep | A “brute force” computational technique used to explore the behavior of a system by systematically varying input parameters over a range of values and observing the corresponding outputs, often to understand how changes in parameters affect the system's behavior or performance. |
| Remediation | As used here, a reversion to pre-intervention conditions; the reversal of the changes imposed on the natural environment by genetic biocontrol. |
| Replacement technology | As used here, a public health intervention approach leveraging genetic biocontrol that introduces modified or transgenic organisms to a wild population for the purpose of wholly replacing the vector competent population with organisms incapable of spreading disease. |
| Resource conservation | The practice of efficiently managing and applying available resources (e.g., funds, materials, labor, time), here in the context of optimizing health, economic, or environmental objectives. |
| Simulation algorithm | A computational method used to model and simulate processes or systems by generating data that mimics their behavior over time or under various conditions. |
| Solution algorithm | A mathematical method or program that finds the solution to a problem. |
| Solution space | The set of all possible solutions (i.e., combinatorial combinations) to a given problem. |
| Solver | A program or algorithm that finds solutions to computational problems, e.g. by applying mathematical optimization or search techniques. |
| Stochastic | A process that involves randomness or uncertainty, where outcomes are not deterministic and may follow probabilistic distributions. |
| Stochastic optimization | A mathematical method for finding the “best” (“optimal”) solution to a problem according to a set of rules or algorithms, incorporating randomness or uncertainty. |
| Struct | A custom composite data type that can contain different types of variables (e.g., integers, floats, strings, other structs, etc.) and facilitates the management and manipulation of data in an organized manner. Short for “structure.” |
| Suppression technology | As used here, a public health intervention approach leveraging genetic biocontrol that introduces modified or transgenic organisms to a wild population for the purpose of suppressing (reducing) the vector competent population and thus lowering disease risk. |
| Working environment | The software and settings that a user interacts with to develop, test, or use computer programs; it includes the integrated development environment (IDE), text editors, operating system, libraries, and other tools necessary for coding and executing software. |

**References**

1. Hien, N. T. *et al.* Environmental factors influence the local establishment of Wolbachia in Aedes aegypti mosquitoes in two small communities in central Vietnam [version 2; peer review: 2 approved]. *Gates Open Research* **5**, (2021).

2. Dong, Z. *et al.* Heatwaves in Southeast Asia and their changes in a warmer world. *Earth’s Future* **9**, e2021EF001992 (2021).

3. The World Bank Group. Climate Change Knowledge Portal. The World Bank Group (2021).

4. Hay, D. C. *Data Model Patterns: Conventions of Thought*. (Dorset House Pub., 1996).

5. Sandve, G. K., Nekrutenko, A., Taylor, J. & Hovig, E. Ten Simple Rules for Reproducible Computational Research. *PLOS Computational Biology* **9**, e1003285 (2013).

6. Gentleman, R. C. *et al.* Bioconductor: Open software development for computational biology and bioinformatics. *Genome Biology* **5**, R80 (2004).

7. Tanaka, H., Stone, H. A. & Nelson, D. R. Spatial gene drives and pushed genetic waves. *Proceedings of the National Academy of Sciences* **114**, 8452–8457 (2017).

8. Huestis, D. L. *et al.* Windborne long-distance migration of malaria mosquitoes in the Sahel. *Nature* **574**, 404–408 (2019).

9. Resnick, S. I. *Adventures in Stochastic Processes*. Springer Science & Business Media (1992).

10. Heimpel, G. E. & Mills, N. J. *Biological Control*. Cambridge University Press (2017).

11. Gurney, W. S. C., Nisbet, R. M. & Lawton, J. H. The systematic formulation of tractable single-species population models incorporating age structure. *The Journal of Animal Ecology* 479–495 (1983).

12. Hurtado, P. J. & Kirosingh, A. S. Generalizations of the ‘Linear Chain Trick’: incorporating more flexible dwell time distributions into mean field ODE models. *Journal of mathematical biology* **79**, 1831–1883 (2019).

13. Schneider, S. M. & Ferris, H. Estimation of stage-specific developmental times and survivorship from stage frequency data. *Researches on population ecology* **28**, 267–280 (1986).

14. Wu, S. L. *et al.* MGDrivE 2: A simulation framework for gene drive systems incorporating seasonality and epidemiological dynamics. *PLOS Computational Biology* **17**, e1009030 (2021).

15. Rossi, M. M., Ólivêr, L. & Massad, E. Modelling the implications of temperature on the life cycle of Aedes aegypti mosquitoes. in *Ecological Modelling Applied to Entomology* 81–107 (Springer, 2014).

16. Carvalho, D. O. *et al.* Suppression of a field population of Aedes aegypti in Brazil by sustained release of transgenic male mosquitoes. *PLoS neglected tropical diseases* **9**, e0003864 (2015).

17. Sánchez C, H.M., Wu, S.L., Bennett, J.B. and Marshall, J.M., 2020. MGDrivE: A modular simulation framework for the spread of gene drives through spatially explicit mosquito populations. *Methods in Ecology and Evolution*, *11*(2), pp.229-239.

18. Sánchez C., H. M., Wu, S. L., Bennett, J. B. & Marshall, J. M. MGDrivE: A modular simulation framework for the spread of gene drives through spatially explicit mosquito populations. *Methods in Ecology and Evolution* **11**, 229–239 (2020).

19. Vásquez, V. N., Kueppers, L. M., Rašić, G. & Marshall, J. M. wMel replacement of dengue-competent mosquitoes is robust to near-term climate change. *Nature Climate Change* (2023).

20. Rackauckas, C. & Nie, Q. Differentialequations. jl–a performant and feature-rich ecosystem for solving differential equations in julia. *Journal of Open Research Software* **5**, (2017).

21. Waechter, A. & Laird, C. Ipopt: Documentation. https://coin-or.github.io/Ipopt/ (2022).

22. Mogensen, P.K., Carlsson, K., Villemot, S., Lyon, S., Gomez, M., Rackauckas, C., Holy, T., Widmann, D., Kelman, T., Karrasch, D. and Levitt, A. *NLsolve.jl.* https://github.com/JuliaNLSolvers/NLsolve.jl (2020).

23. Dunning, I., Huchette, J. & Lubin, M. JuMP: A modeling language for mathematical optimization. *SIAM review* **59**, 295–320 (2017).

24. Lubin, M. & Dunning, I. Computing in operations research using Julia. *INFORMS Journal on Computing* **27**, 238–248 (2015).

25. Bezanson, J., Edelman, A., Karpinski, S. & Shah, V. B. Julia: A fresh approach to numerical computing. *SIAM review* **59**, 65–98 (2017).

26. Roesch, E., Greener, J.G., MacLean, A.L. *et al.* Julia for biologists. *Nature Methods* **20**, 655–664 (2023). https://doi.org/10.1038/s41592-023-01832-z
