# Supplementary figures and images for: GeneDrive.jl: A decision tool to optimize biological vector control strategies under climate change
Source: PLoS Comput Biol. 2025 Oct 21;21(10):e1013600. doi: 10.1371/journal.pcbi.1013600 (PMC12551955; doi:10.1371/journal.pcbi.1013600)

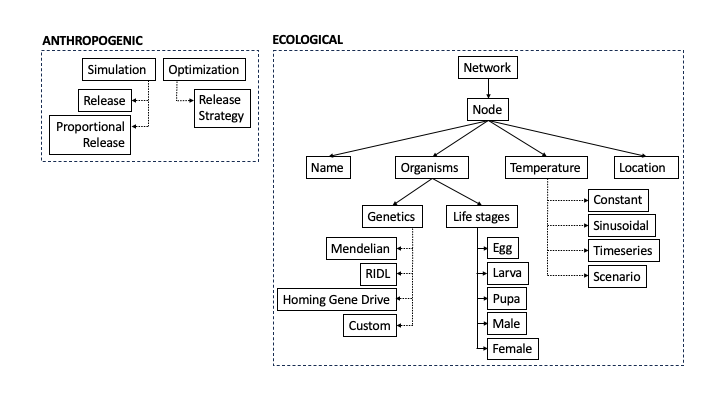

Supplement: S1 Fig — Arrows with dotted lines indicate optional selections, and those with solid lines indicate components that are required to fully specify and store or run a GeneDrive.jl problem. (TIFF) [file pcbi.1013600.s002.tiff]

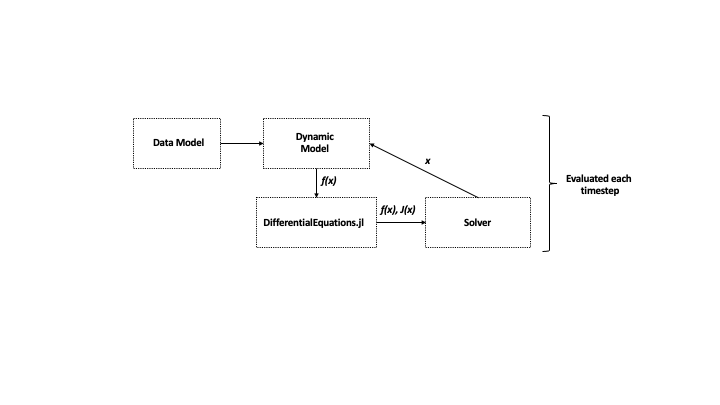

Supplement: S2 Fig — (TIFF) [file pcbi.1013600.s003.tiff]

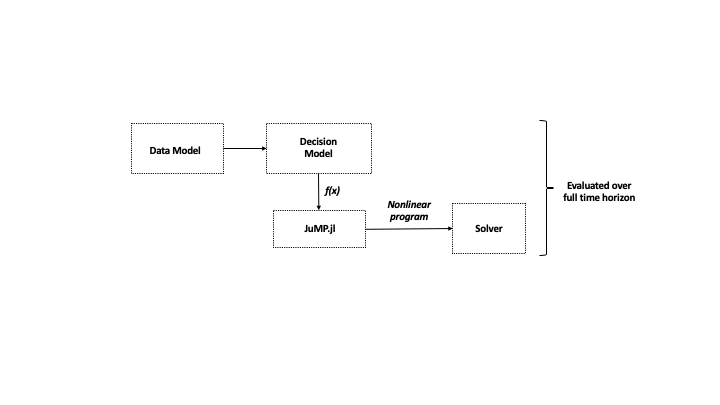

Supplement: S3 Fig — (TIFF) [file pcbi.1013600.s004.tiff]
